# Application of a novel endoscopic device in treatment of gastric bezoars: Pilot study

**DOI:** 10.1055/a-2811-5338

**Published:** 2026-03-06

**Authors:** Jia Xu, Weixing Yang, Xian Zhou, Xuanli Wang, Xia Luo, Haitao Zhang, Mingming Deng, Lei Shi, Xiaowei Tang

**Affiliations:** 1 556508 Department of Gastroenterology, The Affiliated Hospital of Southwest Medical University, Luzhou, China; 2 556508 The Affiliated Hospital of Southwest Medical University, Luzhou, China; 3 556508 Department of Endoscopic Medicine, The Affiliated Hospital of Southwest Medical University, Luzhou, China

**Keywords:** Endoscopy Upper GI Tract, Endoscopic resection (ESD, EMRc, ...), Foreign-bodies

## Abstract

Gastric bezoars, especially phytobezoars, are often challenging to treat due to their large size, hardness, and poor response to conventional endoscopic methods, frequently necessitating surgery. This study presents a novel endoscopic device designed to fragment and remove gastric bezoars as a safer and more efficient alternative. In a retrospective single-center study at the Affiliated Hospital of Southwest Medical University, nine patients with giant gastric bezoars (long axis > 3 cm) were treated using this device between November 2023 and March 2025. The device features two outer sheaths symmetrically attached to the lens body, aligned with a transparent cap, and a yellow zebra guidewire running through the sheaths. The guidewire length can be adjusted to modify the snare size, allowing precise bezoar fragmentation. Patients underwent gastroscopy before and after treatment, with follow-up after 1 month. The device successfully fragmented and removed bezoars in all patients, with an average procedure time of approximately 66 minutes (range 33–113 minutes). No complications occurred and follow-up gastroscopy confirmed complete bezoar removal with significant symptom improvement. The novel endoscopic device demonstrated high efficacy and safety in treating gastric bezoars, offering a promising alternative to surgical intervention.

## Introduction

Gastric bezoars are masses of undigested material that accumulate in the gastrointestinal tract, most commonly in the stomach. These masses are typically composed of indigestible food particles, plant fibers, hair, or medications, and are classified into different types, including phytobezoars, trichobezoars, pharmacobezoars, and lactobezoars 1 . Among these, phytobezoars—composed of plant material such as fibers, fruit skins, and seeds—are the most prevalent. Although some bezoars are asymptomatic, many cause abdominal discomfort, bloating, nausea, or vomiting, and severe cases may lead to gastrointestinal obstruction, bleeding, perforation, or even death 2 .

Treatment of gastric bezoars depends on their size, composition, and severity of associated symptoms. Small bezoars may be managed conservatively with dietary changes, medications, or enzymatic dissolution, whereas larger bezoars often require more invasive treatment. Traditional treatment methods for giant gastric bezoars include endoscopic fragmentation, chemical dissolution with agents such as Coca-Cola or enzymatic preparations, and, in more complicated cases, surgical removal 3 . However, these methods have limitations, particularly in cases involving large, hard bezoars that are resistant to fragmentation or chemical treatments.

Endoscopic fragmentation, which involves use of various tools such as snares and lithotripters, has become the standard approach for non-surgical management of bezoars. Despite its widespread use, endoscopic fragmentation can be challenging for giant bezoars, often requiring prolonged procedure times and sometimes resulting in incomplete removal 4 . Surgical intervention remains an option when endoscopic treatments fail, but surgery is invasive and carries a higher risk of complications, including injury to surrounding structures and longer recovery times.

This study presents a novel endoscopic device for bezoar fragmentation that innovatively employs a guidewire-controlled adjustable snare mechanism that can be precisely adapted to bezoar size for optimal fragmentation. To validate the clinical value of this original device, we prospectively enrolled nine patients with giant gastric bezoars to evaluate its safety, efficacy, and feasibility, aiming to provide a superior therapeutic solution for bezoar management.

## Patients and methods

### Study design and population

This retrospective study was conducted at the Southwest Medical University Affiliated Hospital and included nine patients diagnosed with giant gastric bezoars. Giant bezoars were defined as bezoars with a long axis greater than 3 cm, identified through gastroscopy. The study period extended from November 17, 2023 to March 25, 2025. All patients underwent treatment using a novel endoscopic device specifically designed for fragmentation and removal of large gastric bezoars.

### Novel device specifications

Endoscopic procedure showing fragmentation of a giant gastric bezoar using the novel device.Video 1

**Fig. 1:**
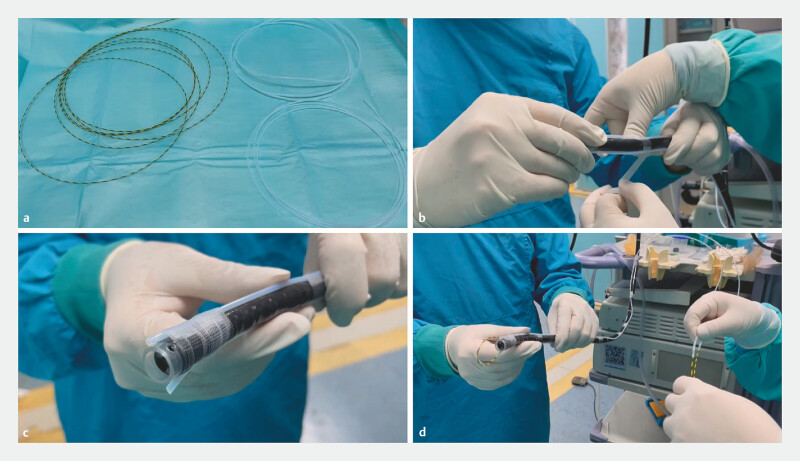
Components of the novel endoscopic bezoar fragmentation device. **a** Two outer sheaths made from injection needle material. **b** The sheaths are symmetrically attached to both sides of the endoscope lens body. **c** The openings of the sheaths align with the transparent distal cap of the endoscope. **d** A yellow zebra guidewire passes through the sheaths.

The novel device consists of two outer sheaths made from injection needle material ( Fig. 1 **a** ), symmetrically attached to both sides of the lens body ( Fig. 1 **b** ), with their openings aligned with the transparent cap of the endoscope ( Fig. 1 **c** ). A yellow zebra guidewire runs through these sheaths, with its length adjustable by the operator from the protruding end ( Fig. 1 **d** ). This adjustability allows snare size to be modified precisely to match the size of the bezoar, facilitating efficient fragmentation. Once adjusted, the snare is used to fragment the bezoar into smaller, manageable pieces, which are then removed in stages.

**Fig. 2:**
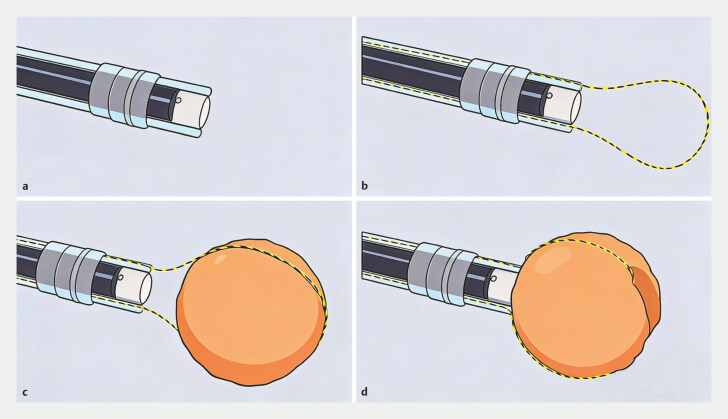
Step-by-step schematic of the bezoar fragmentation procedure using the novel endoscopic device. **a** Initial assembly of the endoscope with the bilateral sheaths. **b** Insertion of the yellow zebra guidewire through the sheaths. **c** Adjustment of the snare loop around the bezoar according to its diameter. **d** Retraction of the guidewire tightens the snare, fragmenting the bezoar through combined mechanical force and coordinated impact from the transparent cap.

The schematic illustration ( Fig. 2 ) demonstrates step-by-step operation of the endoscopic device specifically designed for bezoar fragmentation. Initially, the endoscope is assembled with two outer sheaths ( Fig. 2 **a** ). Subsequently, a yellow zebra guidewire is installed through the sheaths ( Fig. 2 **b** ). Once positioned, the snare is carefully adjusted to encircle the bezoar according to its specific size ( Fig. 2 **c** ). Fragmentation was performed intragastrically by manual retraction of the guidewire, which tightened the snare against the bezoar, aided by mechanical counter-pressure from the transparent cap. No external mechanical winch or traction device was used ( Fig. 2 **d,** Video 1 ). The distal transparent cap played a critical role by stabilizing the bezoar to provide mechanical counter-pressure during snare tightening, protecting the endoscopic lens from debris, and maintaining an optimal viewing field.

### Patient selection criteria

Inclusion criteria for the study were: 1) patients diagnosed with giant gastric bezoars (bezoars with a long axis greater than 3 cm); 2) patients who underwent treatment with the novel endoscopic device; and 3) patients who had complete clinical and follow-up data available. Exclusion criteria included patients with contraindications to endoscopy or those who had previously undergone surgical bezoar removal.

### Clinical procedures

Prior to treatment, all patients underwent gastroscopy to confirm the diagnosis and assess the size and location of the bezoar. The procedure was performed under general anesthesia with nasal cannula oxygenation for respiratory support. During treatment, the adjustable snare was used to fragment the bezoar, with operation time recorded for each patient.

Post-procedure, all patients underwent follow-up gastroscopy 1 month later to assess for complete removal of the bezoar and monitor for any complications such as perforation, bleeding, or residual bezoar fragments. Procedure success was defined as complete removal of the bezoar without any complications. To prevent mucosal injury from guidewire slippage, all manipulations were performed under direct endoscopic visualization. The transparent cap stabilized the bezoar during snare engagement. Fragmentation was then achieved by slowly and steadily retracting the guidewire, applying controlled force between the tightening snare and the cap. No wire-related mucosal injuries occurred.

## Case description

**Fig. 3:**
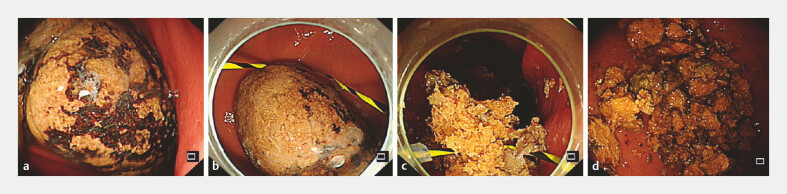
Endoscopic images from a clinical case demonstrating bezoar fragmentation using the novel device. **a** Gastroscopy revealed a large gastric bezoar measuring approximately 4 × 10 cm. **b** The snare was carefully adjusted to encircle the bezoar. **c** Retraction of the guidewire, combined with forward pressure from the transparent cap, initiated fragmentation. **d** The bezoar was successfully broken into smaller fragments, all of which were removed endoscopically.

A 33-year-old woman presented with upper abdominal pain lasting over 5 months, which worsened significantly in the past month. Gastroscopy revealed a gastric bezoar measuring approximately 4 × 10 cm ( Fig. 3 **a** ). The endoscopic fragmentation procedure was performed using a snare device guided by a transparent cap and zebra guidewire. The snare was precisely adjusted and tightened around the bezoar ( Fig. 3 **b** ). Through coordinated retraction of the guidewire and targeted impact from the transparent cap, the bezoar was rapidly fragmented into manageable pieces ( Fig. 3 **c, d** ). These fragments were subsequently removed without complications. The patient experienced complete symptom resolution and follow-up endoscopy confirmed absence of residual bezoar fragments.

## Results

Demographic information about, clinical presentation of, and outcomes for the nine patients treated with the novel endoscopic device are detailed in Table 1 . The cohort comprised five male and four female patients with an average age of 55.4 years (range from 15 to 78 years). Abdominal pain was the most common presenting symptom, reported by all but one patient who presented with melena. None of the included patients had significant medical or familial histories pertinent to formation of bezoars.

**Table:** **Table 1** Clinical characteristics of and surgical information from included patients with gastric bezoars.

No.	Sex	Age (year)	Symptoms	Bezoar diameter (cm)	Operation time (min)	Adverse events	Postoperative length of hospital stay (day)
1	Female	71	Abdominal pain	5×15	113	No	2
2	Female	33	Abdominal pain	4×10	85	No	2
3	Male	78	Abdominal pain	5×9	81	No	6
4	Male	50	Abdominal pain	4×5	53	No	1
5	Female	51	Abdominal pain	4×5	38	No	1
6	Male	15	Abdominal pain	4×4	33	No	3
7	Male	71	Abdominal pain	4×8	52	No	2
8	Male	53	Abdominal pain	3×4	34	No	3
9	Female	77	Abdominal pain	6×10	105	No	3

Endoscopic examination prior to treatment revealed gastric bezoars with an average size of approximately 4.3 cm × 7.8 cm, ranging from 3 cm × 4 cm to as large as 5 cm × 15 cm. All bezoars were successfully fragmented into smaller segments (< 1 cm) using the novel adjustable snare device in a single session. Procedure time, defined as duration between endoscope insertion and withdrawal (excluding anesthesia induction), was documented for each case, averaging 66 minutes.

No procedure complications or adverse events (AEs), including gastrointestinal injury, bleeding, or residual bezoar fragments, occurred in any patient. Postoperative hospital stays ranged from 1 to 6 days, with an average stay of approximately 2.5 days. Follow-up gastroscopy performed approximately 1 month post-procedure confirmed complete bezoar removal and recovery without residual fragments or further complications.

## Discussion

Gastric bezoars, particularly giant phytobezoars, pose a significant therapeutic challenge due to their size and density, which often resist chemical dissolution and standard endoscopic removal. Although surgery remains an option, its associated risks and recovery time underscore the need for safer, efficient endoscopic alternatives. To address this, we developed a simple yet innovative endoscopic device consisting of only two injection needle sheaths and a guidewire, which can be easily assembled with a standard endoscope. This design lowers cost, simplifies setup, and enhances accessibility—especially in resource-limited settings lacking specialized equipment. Our pilot trial confirms the technique's clinical efficacy, cost-effectiveness, and potential as a widely applicable minimally invasive intervention.

The study demonstrated complete endoscopic success in all patients, achieving effective single-session fragmentation regardless of bezoar size or texture—a notable outcome particularly for bezoars > 5 cm or of dense composition, for which conventional tools often lead to prolonged or incomplete procedures 5 . This efficacy is attributed to device design: Its dual outer sheath system and transparent cap enhance endoscopic control and visualization by providing stable counter-pressure and ensuring even force distribution, which contributed to absence of AEs and distinguishes it from existing methods 6 . Consequently, patients experienced prompt resolution of symptoms such as abdominal discomfort and nausea, supported by follow-up gastroscopy confirming complete bezoar removal without mucosal injury, underscoring the intervention’s clinical effectiveness.

It is important to contextualize our findings alongside a significant contemporary study on endoscopic bezoar management. Toka et al. prospectively evaluated a "hand-made bezoaratome”—a guidewire-based snare assembled from a mechanical lithotripter sheath—in 37 patients with large phytobezoars (≥ 50 mm) 7 . Although both techniques achieved 100% fragmentation success, key differences exist. Median procedure time in the Toka et al. report was shorter (~14 min vs. our 66 min), likely because our cohort had larger bezoars and we pursued complete clearance to fragments < 1 cm in a single session, whereas their protocol allowed fragments < 15 mm to pass spontaneously. Our device, with its fixed sheaths and transparent cap, may enable more controlled, complete clearance during one procedure, potentially reducing risk of distal fragment obstruction. This comparison underscores the value of innovative endoscopic approaches for giant bezoars and provides a useful benchmark for newer techniques like ours.

Compared with previously documented methods, this approach appears to offer several clinical advantages. Chemical dissolution using agents such as coke or cellulase, although noninvasive, often requires prolonged administration and carries unpredictable efficacy, especially for large or calcified bezoars 8 . Moreover, certain patients may not tolerate such therapies due to underlying conditions such as diabetes, gastric ulceration, or electrolyte imbalance. Mechanical endoscopic methods—using forceps, baskets, or lithotripters—have been more widely adopted, but procedure success remains operator-dependent and may require considerable time 9 . Published series have shown that in some cases, endoscopic fragmentation may fail altogether, necessitating surgical extraction 10 . In contrast, the method described in this study demonstrated a high success rate within a relatively short procedure window, without the need for adjunctive pharmacological measures or surgical backup. Furthermore, the procedure was well-tolerated, with no mucosal trauma, bleeding, or infection observed. All patients, including those with bezoars up to 15 cm, were discharged within days, suggesting the approach may improve recovery efficiency and reduce resource use.

Nonetheless, this study also has limitations. Its retrospective design may introduce selection bias because only successfully treated cases were included. Future studies should encompass a broader range of presentations, including failures. In addition, although our sample size is typical for early-phase research, larger cohorts are needed to confirm generalizability, particularly for patients with altered anatomy (e.g., post-surgery) or bezoars located beyond the stomach.

The device's increased outer diameter due to the attached sheaths raises a theoretical risk of mucosal injury during insertion. However, no such injuries occurred in our series. This is likely attributable to cautious insertion technique, the sheaths' smooth, edgeless material, and device operational stability, which minimizes traumatic movement. Gentle technique and training are advised and future designs could aim for a lower profile to further address this concern.

Moreover, the study's lack of a control group limits direct comparison with standard therapies, underscoring the need for prospective randomized comparisons with conventional fragmentation or chemical dissolution. Longer follow-up beyond 1 month is also required to assess recurrence risk in patients with predisposing factors. Operator experience likely contributed to consistent outcomes, suggesting that standardized training would be beneficial for broader adoption.

Looking forward, this method shows promise for other intraluminal masses, such as pharmacobezoars or food obstructions. Future device iterations could integrate suction or digital tension control to enhance utility. Although initial costs exist, reduced procedure time, avoidance of repeat sessions or surgery may offer long-term economic advantages, warranting formal cost-effectiveness analysis—especially in resource-limited settings.

## Conclusions

In conclusion, our findings support the utility of this novel endoscopic device, highlighting its precision, efficiency, safety, and significant economic advantages. Because of its simplicity, cost-effectiveness, and ease of deployment, the device has the potential for broad implementation in resource-limited settings.
